# Involvement in Home Cooking Activities Is Inversely Associated with Emotional Problems Among Brazilian Adolescents

**DOI:** 10.3390/nu18213606

**Published:** 2026-10-30

**Authors:** Guilherme José Silva Ribeiro, Rafaela Mara Silva Fonseca, Rafaela Nogueira Gomes de Morais, André de Araújo Pinto, Bruna Clemente Cota, Mariana de Santis Filgueiras, Juliana Farias de Novaes

**Affiliations:** 1 Graduate Program in Nutrition Science, Department of Nutrition and Health, Federal University of Viçosa, Viçosa 36570-900, MG, Brazil; rafaela.fonseca@ufv.br (R.M.S.F.); rafaela.morais@ufv.br (R.N.G.d.M.); mariana.filgueiras@ufv.br (M.d.S.F.);; 2 Department of Physical Education, State University of Roraima, Boa Vista 69306-530, RR, Brazil; andre.pinto@uerr.edu.br; 3 Institute of Collective Health, Federal University of Bahia (UFBA), Salvador 40170-115, BA, Brazil; brunacota@ufba.br

**Keywords:** mental health, cooking, loneliness, sadness, psychological distress, suicide, adolescent

## Abstract

Background/Objectives: Involvement in home cooking is associated with a healthy diet. However, its relationship with emotional problems has not been investigated among adolescents, including loneliness, sadness, psychological stress, and suicidal ideation. The objective of this study was to investigate the association between involvement in home cooking and these emotional problems among Brazilian adolescents. Methods: This is a cross-sectional study that evaluated 1032 participants aged 14 to 19 years enrolled in nine public state schools in Boa Vista, Roraima, Brazil, 2025. The “Involvement in Home Cooking” questionnaire for Brazilian adolescents was used. Emotional problems (loneliness, sadness, psychological stress, and suicidal ideation) were assessed using validated questionnaires. Poisson regression models with robust variance were used to identify the association between the variables studied, considering potential confounding factors. Results: Adolescents who “always” participated in meal planning had a lower prevalence of loneliness; those who “sometimes” participated in food selection/purchasing had lower prevalences of sadness, loneliness, and psychological stress, while those who “always” participated in this activity had lower prevalences of loneliness and suicidal ideation; and those who “sometimes” participated in meal preparation for the family had lower prevalences of sadness, loneliness, and psychological stress, while those who “always” participated in it had lower prevalences of sadness and loneliness (*p* < 0.05). Conclusions: Involvement in home cooking was inversely associated with emotional problems among Brazilian adolescents. This association reinforces the importance of considering domestic culinary routines in strategies aimed at promoting adolescents’ emotional well-being.

## 1. Introduction

Emotional problems can be understood as manifestations of psychological distress encompassing different aspects of mental health, including sadness, loneliness, psychological stress, and suicidal ideation, among others. Although related, these indicators reflect different experiences: sadness involves negative affect [1], loneliness is associated with the perceived inadequacy of social relationships [2], psychological stress reflects difficulties in coping with demands perceived as excessive [3], and suicidal ideation refers to thoughts about suicide [4]. These conditions have been associated with psychopathology [5], immunometabolic syndromes [6,7], and suicide attempts [8]. Such experiences are relevant across the lifespan, including during adolescence [9].

In Brazil and Latin America, recent data reflect a high burden of emotional problems. Approximately 12.1% of adolescents report loneliness in Latin America [10], while in Brazil, 28.9% report frequent feelings of sadness [11] and 21.0% experience psychological stress [12]. Crucially, the prevalence of suicidal ideation among Brazilian adolescents aged 14–19 years rose from 11.2% in 2006 to 26.7% in 2022 [13]. In this context, identifying factors associated with better emotional health among adolescents is a public health goal, particularly in countries such as Brazil.

Involvement in home-based culinary activities, such as participating in meal planning, food selection and purchasing, and preparing meals for the family, involves cognitive, psychosocial, and behavioral processes that may be related to adolescents’ emotional health [14]. For example, participation in these activities may foster autonomy and self-efficacy by providing opportunities to develop practical skills, make decisions, and experience a sense of competence [15,16,17,18,19]. Additionally, culinary activities may encourage family interaction and social connectedness, strengthening family relationships and reducing psychosocial difficulties [20,21]. Furthermore, involvement in meal planning and preparation may be associated with healthier eating habits, which may contribute to emotional well-being [20,22].

Previous studies have reported associations between adolescents’ involvement in cooking activities and emotional well-being. Utter et al. (2016) [22], in a study conducted in New Zealand, found that cooking skills and behaviors were associated with a lower likelihood of depressive symptoms and better mental well-being. More recently, Castillo-Miñaca et al. (2025) [23], in Spain, found that more frequent involvement in meal preparation and food shopping was associated with fewer psychosocial difficulties, including internalizing problems.

In Brazil, Neves et al. (2023) [24] investigated adolescents’ involvement in home cooking activities, including meal planning, food selection and purchasing, and meal preparation for the family, reporting greater involvement among girls than boys. However, evidence remains limited regarding the associations between these specific domains of involvement and distinct emotional health outcomes during adolescence. In particular, the relationship between adolescents’ involvement in home cooking activities and suicidal ideation remains insufficiently investigated. Evidence from Brazilian adolescents is also scarce, particularly among those living in the Amazon region.

This study seeks to address these gaps by investigating the associations between three domains of home cooking involvement (meal planning, food selection and purchasing, and meal preparation) and four emotional health outcomes (sadness, loneliness, psychological stress, and suicidal ideation) among adolescents in Boa Vista, Roraima, Brazil. We hypothesized that greater involvement in each of these home cooking involvement would be associated with a lower prevalence of the outcomes investigated.

## 2. Materials and Methods

### 2.1. Study Design and Participants

This cross-sectional study included a large school-based sample of adolescents enrolled in nine public schools across all nine educational districts in Boa Vista, Roraima, Brazil, from March to May 2025. All procedures and questionnaires followed the Declaration of Helsinki and were approved by the Human Research Ethics Committee of the State University of Roraima (protocol no. 7264601/2024).

In 2024, the local education system reported 28,745 high school students distributed across 58 schools organized into nine educational districts. The sample size was calculated based on this population, assuming a 50% prevalence, a 95% confidence level, a 5% margin of error, and a design effect of 2.0 [25]. An additional 20% was added to account for potential losses, resulting in a minimum required sample of 910 adolescents. Although the primary sample size calculation was designed for prevalence estimation, a post hoc sensitivity power analysis indicated that the final analytical sample (*n* = 1032; effective sample size, *n* = 516, assuming a design effect of 2.0) provided 80% power at a significance level of 5% to detect minimum prevalence ratios ranging from 1.24 to 1.38 across the primary emotional outcomes.

A two-stage school-based sampling procedure was used. The nine official educational districts were defined as strata, and the target number of participants in each district was determined proportionally to its high school student enrollment. In the first stage, the school with the largest student enrollment in each district was purposively selected, resulting in nine participating schools. Thus, all nine educational districts were covered, with one participating school per district. In the second stage, classes from the first, second, and third years of high school were randomly selected within each participating school until the target sample size for each district was reached, with additional classes selected when necessary [26]. All eligible students enrolled in the selected classes were invited to participate.

Eligibility criteria included students aged 14 to 19 years who were regularly enrolled in school. Pregnant females and adolescents with cognitive impairments that precluded questionnaire completion were excluded. The initial sample comprised 1051 adolescents. The final analytical sample comprised 1032 participants, as some individuals did not meet the eligibility criteria, including age above 19 years (*n* = 1), pregnancy (*n* = 6), or inability to complete the questionnaire independently (*n* = 12).

A pilot study was conducted prior with 67 adolescents to the main investigation to assess the clarity, comprehensibility, and applicability of the data collection instruments, as well as the feasibility of the methodological procedures. Questionnaires were administered at two distinct time points with a 3-day interval (Tuesday and Friday) to evaluate test–retest reliability. Participants in the pilot study were not included in the final study sample.

Written informed consent was obtained from parents or legal guardians of participants under 18 years of age, and written assent was obtained from the adolescents.

### 2.2. Variables

#### 2.2.1. Emotional Problems

To assess emotional problems, adolescents responded to questions addressing sadness, loneliness, psychological stress, and suicidal ideation using validated questionnaires [27,28]. Sadness was assessed using an item from the Brazilian National School Health Survey (PeNSE), conducted by the Brazilian Institute of Geography and Statistics in partnership with the Ministry of Health [27]. Loneliness and psychological stress were assessed using items from the COMPAC questionnaire (Adolescent Behaviors of Santa Catarina), specifically from the “Health Perception and Preventive Behavior” section [28].

The questions were as follows: “In the past 30 days, how often have you felt sad?” [27] and “In the past 12 months, how often have you felt lonely?” [28]. Response options ranged from “never,” “rarely,” “sometimes,” “often,” to “always.” Feelings of sadness and loneliness were considered present when responses were “often” or “always.”

Psychological stress was assessed using the question “How often do you describe your life as stressful?” [28]. Response options were as follows: (1) rarely stressed, living very well; (2) sometimes stressed, living reasonably well; (3) almost always stressed, frequently facing problems; and (4) excessively stressed, with difficulty dealing with daily life. Adolescents were classified as experiencing psychological stress when they selected “almost always stressed” or “excessively stressed.”

Ordinal responses were dichotomized following established protocols from the original population surveys [27,28] to isolate clinically relevant high-frequency symptoms (‘often’/’always’ and ‘almost always’/’excessively’) and to avoid small subgroup sample sizes in multivariable modeling, facilitating epidemiological interpretation.

Suicidal ideation was assessed using an item from the Brazilian-adapted version of the Youth Risk Behavior Survey (YRBS): “In the past 12 months, have you seriously considered attempting suicide?” Response options were “yes” or “no.”

#### 2.2.2. Involvement in Home Cooking

Involvement in home cooking was assessed using three items from the Questionnaire for the Assessment of Adolescents’ Involvement in Home Cooking, developed by Neves et al. (2023) (EVA-JF study) [24]. The original questionnaire content was based on the Dietary Guidelines for the Brazilian Population and underwent formal content validation by an expert panel of six nutritional epidemiologists, as well as psychometric testing demonstrating high internal consistency (Cronbach’s (Cronbach’s α = 0.821). The items assessed adolescents’ participation in (1) family meal planning, (2) food selection and/or purchasing, and (3) meal preparation. These items were selected because they represent practical involvement in everyday domestic culinary activities, reflecting behaviors embedded in family and social dynamics, which are potentially relevant for understanding outcomes related to emotional well-being. In the present study, the questionnaire demonstrated high reproducibility, with Cohen’s Kappa coefficients (κ) ranging from 0.64 to 1.00. The mean coefficient was κ = 0.84, which, according to the classification proposed by Landis & Koch, (1997) [29], indicates almost perfect agreement in test–retest reliability. These findings support the instrument’s stability and reliability for assessing adolescent engagement in home cooking.

Questions:

(A) How often do you participate in meal planning for your family?
(B) How often do you participate in selecting and/or purchasing food for your family? In food stores: bakeries, grocery stores, markets, butchers, greengrocers, fairs, etc.
(C) How often do you participate in preparing meals for your family? Before: separating food and culinary ingredients, cutting and seasoning, cooking, stirring the food, controlling the time, setting the table, etc. After: clearing the table, sanitizing crockery and cutlery, organizing the kitchen, etc.

The answer options for all questions were never (reference group in multivariable models), sometimes, and always.

### 2.3. Covariates

The covariates were collected with adolescents by self-administered questionnaires. Sociodemographic data were evaluated considering school shift (morning, afternoon, or evening), sex (male or female), age (years), race/skin color (yellow, white, brown, black, and indigenous), and family income (up to 2 minimum wages, 3 to 5 minimum wages, or ≥6 minimum wages), considering one minimum wage equivalent to US$304.00 in 2025.

Body mass index (BMI) was classified according to international age- and sex-specific cutoff points [30], with the nutritional status was grouped into two categories: underweight/normal weight and overweight/obesity, based on self-reported weight and height provided by the adolescents.

Information on physical activity (insufficiently <60 min/day and sufficiently ≥60 min/day) [31] and screen time (≤2 h/day and >2 h/day) [32] were evaluated.

### 2.4. Statistical Analysis

Exposure: Involvement in home cooking.
Outcome: Emotional problems (sadness, loneliness, psychological stress and suicidal ideation).

The three domains of involvement in home cooking and the four emotional outcomes were prespecified before the statistical analyses. Participants’ characteristics were presented as absolute and relative frequencies and as means ± standard deviations, as appropriate. Pearson chi-square tests adjusted for the complex survey design were used to compare the prevalence of emotional problems across categories of involvement in home cooking. To estimate associations between exposure and outcome variables, Poisson regression models with robust variance were employed, and prevalence ratios (PRs) with corresponding 95% CIs were calculated. To account for the complex survey design, all analyses incorporated post-stratification sampling weights, stratification by school district, and clustering at the classroom level. Statistical models were adjusted for sociodemographic variables (age, sex, self-reported race/skin color, school shift, and family income), lifestyle factors (physical activity and screen time), and nutritional status. Interactions between involvement in home cooking and age, sex, and skin color were assessed by including interaction terms in the adjusted Poisson regression models. All analyses were conducted using Stata version 17 (StataCorp LP, College Station, TX, USA), considering the significance level of 5%.

## 3. Results

Among the 1032 study participants, the mean age was 16 years and 50.5% were male. Most adolescents self-identified as brown (53.8%), 44.9% attended school in the afternoon shift, and 59.2% reported a family income of up to two Brazilian minimum wages. Regarding lifestyle, 59.5% of adolescents were insufficiently active and 98.3% spent more than 2 h per day in front of screens. In addition, 40.5% presented overweight or obesity. Regarding emotional problems, 32.1% of adolescents reported sadness, 35.6% loneliness, 49.1% psychological stress, and 32.4% suicidal ideation (Table 1).

It was observed that 39.4% and 32% of adolescents reported participating “sometimes” and “always,” respectively, in meal planning for the family. Regarding food selection/purchase, 34.2% and 33.8% reported participating “sometimes” and “always”, respectively. Considering meal preparation for the family, 36.2% and 35.1% of adolescents reported participating “sometimes” and “always,” respectively (Table 2).

The prevalence of loneliness was highest among adolescents who “never” participated in meal planning and food selection/purchasing. In addition, the prevalence of psychological stress was highest among adolescents who “never” participated in food selection/purchasing (*p* < 0.05) (Table 3).

In adjusted multivariable models, we observed the following results for emotional problems in adolescents according to their involvement in home cooking (Table 4):

Meal planning. A lower prevalence of loneliness was observed among adolescents who always participated in meal planning (PR = 0.61, 95% CI: 0.46–0.79; *p* = 0.001). A significant linear trend was observed for loneliness (*p* for trend < 0.001).

Food selection/purchasing. Lower prevalences of sadness (“sometimes”: PR = 0.75, 95% CI: 0.60–0.94; *p* = 0.017), loneliness (“sometimes”: PR = 0.71, 95% CI: 0.56–0.89; *p* = 0.005; “always”: PR = 0.76, 95% CI: 0.64–0.91; *p* = 0.004), psychological stress (“sometimes”: PR = 0.78, 95% CI: 0.65–0.93; *p* = 0.007), and suicidal ideation (“always”: PR = 0.93, 95% CI: 0.87–0.99; *p* = 0.046) were observed among adolescents who participated in food selection/purchasing. Significant linear trends were observed for loneliness (*p* for trend = 0.005) and suicidal ideation (*p* for trend = 0.047).

Meal preparation. A lower prevalence of sadness (“sometimes”: PR = 0.81, 95% CI: 0.66–0.99; *p* = 0.047; “always”: PR = 0.83, 95% CI: 0.70–0.99; *p* = 0.044), loneliness (“sometimes”: PR = 0.72, 95% CI: 0.55–0.94; *p* = 0.017; “always”: PR = 0.77, 95% CI: 0.60–0.97; *p* = 0.034), and psychological stress (“sometimes”: PR = 0.83, 95% CI: 0.73–0.94; *p* = 0.005) was observed among adolescents who participated in meal preparation. No statistically significant linear trends were observed for any outcome in this domain (*p* for trend ≥ 0.05).

## 4. Discussion

Our findings showed that associations between involvement in home cooking and emotional problems varied according to the culinary activity and outcome examined. Adolescents who “always” participated in meal planning had a lower prevalence of loneliness; those who “sometimes” participated in food selection/purchasing had lower prevalences of sadness, loneliness, and psychological stress, while those who “always” participated in this activity presented lower prevalences of loneliness and suicidal ideation; and those who “sometimes” participated in meal preparation for the family had lower prevalences of sadness, loneliness, and psychological stress, while those who “always” participated in it had lower prevalences of sadness and loneliness. However, these associations did not follow a dose-dependent pattern across the “sometimes” and “always” categories of involvement. Similar findings were observed by cross-sectional studies where the adolescent involvement in home cooking was associated with better emotional health, including lower internalizing problem scores [23], fewer depressive symptoms, and improved mental well-being [22].

The observed associations may be related to three potential pathways, although they were not directly assessed in the present study. First, participation in meal planning and preparation may contribute to the development of autonomy, self-efficacy, decision-making, and emotional self-regulation [14,16,20,32,33,34,35,36]. These skills have been associated with more favorable mental health indicators, including lower levels of loneliness among adolescents [37]. Second, culinary activities involving food selection and purchasing may provide opportunities for social interaction and the development of a sense of competence [38,39,40]. Similarly, participation in meal preparation and family meals has been associated with higher life satisfaction, fewer psychosomatic complaints, fewer depressive symptoms, and a lower prevalence of suicidal behaviors [41,42]. Third, participation in food preparation may be associated with greater cooking skills and better diet quality [20,43,44,45]. Healthier dietary patterns have been linked to more favorable emotional outcomes, including lower psychological stress and higher life satisfaction [41,46], suggesting that diet quality may be another potential pathway.

Associations with sadness were observed among adolescents who reported “sometimes” or “always” participating in meal preparation. An inverse association was also observed between suicidal ideation and involvement in food selection and purchasing. Emotional well-being and suicidal ideation are major public health concerns and these associations differed according to the type and frequency of participation in household food-related activities Although these associations were small in magnitude, their relevance to public health should not be dismissed solely based on effect size. This is particularly important in adolescence, when Even modest associations may warrant attention in this context, although their practical implications require further investigation. Future longitudinal studies are needed to clarify the temporality of these associations, investigate potential underlying mechanisms, and determine whether these findings are consistent across different adolescent populations.

Although few studies have investigated the relationship between involvement in home cooking activities and adolescent mental health, the domestic culinary environment may be relevant to understanding factors associated with psychological well-being [47]. Participation in meal-related activities, such as meal planning, food selection and purchasing, and meal preparation, may provide opportunities for family interaction and emotional support. Therefore, more studies are necessary since there is currently insufficient evidence to recommend their incorporation into clinical practice or public health programs as a strategy to improve adolescent mental health.

Furthermore, these findings should be considered in the cultural and socioeconomic context of adolescents’ participation in home cooking activities. In Brazil, such involvement may reflect family traditions, gender-related expectations, and the distribution of household responsibilities. While some adolescents may participate in meal preparation as an opportunity for learning and family bonding, others may do so out of necessity, in response to domestic demands or limited access to convenient and affordable food alternatives. These distinct circumstances may shape the subjective meaning of culinary involvement and its relationship with mental health. However, because the present study did not investigate adolescents’ motivations or family dynamics, these explanations remain hypothetical. In this regard, further longitudinal and interventional studies are needed to clarify temporal relationships and underlying mechanisms and to determine whether promoting involvement in home cooking activities can contribute to better mental health outcomes among adolescents.

This study has several limitations. The findings should not be generalized to the broader adolescent population, as the sample consisted exclusively of students enrolled in public state schools in Boa Vista, Roraima, in the Northern region of Brazil. In addition, the cross-sectional design precludes causal inference between the investigated variables. The use of a self-administered questionnaire may have introduced information bias due to misinterpretation of questions or socially desirable responses. Another limitation is the lack of more detailed information regarding the family context, including family functioning, parental support, family composition, frequency of family meals, food security, parental mental health, and the distribution of household responsibilities. These factors may influence both adolescents’ involvement in home cooking and their emotional well-being, potentially contributing to residual confounding. Their assessment could provide a more comprehensive understanding of the associations investigated.

However, this study has notable strengths. To our knowledge, it is one of the few studies to investigate the association between involvement in home cooking and mental health among adolescents in low- and middle-income countries such as Brazil, using data from public state school students in Amazon region that remains underrepresented in epidemiological research. Furthermore, the use of validated instruments and adjustment for relevant confounding variables strengthens the study.

## 5. Conclusions

Adolescents’ involvement in home cooking activities was associated with lower prevalences of emotional health problems, although these associations varied according to the type of culinary activity and the outcome examined. Involvement in food selection and purchasing and meal preparation was associated with lower prevalences of sadness, loneliness, and psychological stress, whereas meal planning was associated with lower prevalence of loneliness. In addition, a lower prevalence of suicidal ideation was observed among adolescents with greater involvement in food selection and purchasing. Domestic culinary engagement may represent a potentially relevant family behavior in the context of adolescent mental health. However, longitudinal and interventional studies are needed to establish temporality and determine whether these associations persist after accounting for family functioning, socioeconomic context, and other potential confounders, before such practices can be recommended as a mental health promotion strategy.

## Figures and Tables

**Table 1:** Sociodemographic, lifestyle, and health characteristics of adolescents. Boa Vista, Roraima, Brazil, 2025.

Variables	n (%)
Sex	
Male	521 (50.5)
Female	511 (49.5)
Age †	16.12 ± 0.99
Skin color/race	
White	218 (21.1)
Black	159 (15.4)
Brown	555 (53.8)
Yellow	38 (3.7)
Indigenous	62 (6.0)
School shift	
Morning	453 (43.9)
Afternoon	463 (44.9)
Evening	116 (11.2)
Monthly household income (US$)	
≤911	614 (59.5)
912–1823	309 (29.9)
≥1824	109 (10.6)
Physically active	
No	614 (59.5)
Yes	418 (40.5)
Screen time (hour/day)	
≤2	18 (1.7)
>2	1.014 (98.3)
Nutritional status	
Underweight/normal weight	614 (59.5)
Overweight/obesity	418 (40.5)
Sadness	
No	701 (67.9)
Yes	331 (32.1)
Loneliness	
No	665 (64.4)
Yes	367 (35.6)
Psychological stress	
No	525 (50.9)
Yes	507 (49.1)
Suicidal ideation	
No	698 (67.6)
Yes	334 (32.4)

Note: n, absolute frequency; %, relative frequency. † Mean ± standard deviation. Conversion from Real for Dollar (R$1.00 = US$0.20) in 2025.

**Table 2:** Adolescent involvement in home cooking: meal planning and preparation, and food selection/purchase. Boa Vista, Roraima, Brazil, 2025.

Involvement in Home Cooking	n (%)
Meal planning	
Never	302 (28.6)
Sometimes	404 (39.4)
Always	326 (32.0)
Food selection/purchasing	
Never	333 (32.0)
Sometimes	359 (34.2)
Always	340 (33.8)
Meal preparation	
Never	306 (28.7)
Sometimes	371 (36.2)
Always	355 (35.1)

Note: n, absolute frequency; %, relative frequency.

**Table 3:** Prevalence of emotional problems according to adolescent involvement in home cooking. Boa Vista, Roraima, Brazil, 2025.

	Emotional Problems							
Involvement in Home Cooking	Sadness n (%)		Loneliness n (%)		Psychological Stress n (%)		Suicidal Ideation n (%)	
	**Yes**	**No**	**Yes**	**No**	**Yes**	**No**	**Yes**	**No**
Meal planning								
Never	113 (37.5)	189 (62.5)	117 (40.4)	185 (59.6)	158 (51.2)	144 (48.8)	95 (30.8)	207 (69.2)
Sometimes	131 (32.1)	273 (67.9)	129 (32.4)	275 (67.6)	191 (45.2)	213 (54.8)	135 (32.2)	269 (67.8)
Always	123 (38.8)	203 (61.2)	85 (26.1)	241 (73.9)	158 (49.7)	168 (50.3)	104 (32.6)	222 (67.4)
*p*-value	0.178		0.029		0.433		0.912	
Food selection/purchasing								
Never	133 (39.4)	200 (60.6)	127 (38.9)	206 (61.1)	175 (51.8)	158 (48.2)	118 (35.0)	215 (65.0)
Sometimes	111 (31.2)	248 (68.8)	97 (28.0)	262 (72.0)	156 (41.5)	203 (58.5)	112 (30.2)	247 (69.8)
Always	123 (37.1)	217 (62.9)	107 (31.3)	233 (68.7)	176 (52.0)	164 (48.0)	104 (30.8)	236 (69.2)
*p*-value	0.146		0.018		0.012		0.454	
Meal preparation								
Never	115 (36.8)	191 (63.2)	115 (38.2)	191 (61.8)	160 (50.0)	146 (50.0)	101 (33.9)	205 (68.1)
Sometimes	122 (32.6)	249 (67.4)	102 (28.5)	269 (71.5)	164 (43.8)	207 (56.2)	110 (29.0)	261 (71.0)
Always	130 (38.2)	225 (61.8)	114 (32.3)	241 (67.7)	183 (51.7)	172 (48.3)	123 (35.0)	232 (65.0)
*p*-value	0.315		0.087		0.126		0.331	

Values are presented as unweighted frequencies (n) and weighted row percentages (%). *p*-values were obtained using design-adjusted Pearson chi-square tests, accounting for sampling weights, stratification by school, and clustering at the classroom level.

**Table 4:** Multivariable models examining the association between involvement in home cooking and emotional problems among adolescents. Boa Vista, Roraima, Brazil, 2025.

Emotional Problems	How Often Do You Participate in the Following Activities for Your Family?								
	Meal Planning			Food Selection/Purchasing			Meal Preparation		
	Never	Sometimes	Always	Neve	Sometimes	Always	Never	Sometimes	Always
Sadness									
Unadjusted model (PR; 95% CI)	Ref.	0.86 (0.67–1.09)	1.03 (0.85–1.26)	Ref.	0.79 (0.62–1.02)	0.94 (0.75–1.18)	Ref.	0.89 (0.69–1.13)	1.04 (0.84–1.28)
*p*-value		0.196	0.724		0.067	0.596		0.318	0.724
Adjusted model (PR; 95% CI)	Ref.	0. 84 (0.69–1.02)	0.91 (0.76–1.07)	Ref.	**0.75 (0.60–0.94)**	0.83 (0.67–1.01)	Ref.	**0.81 (0.66–0.99)**	**0.83 (0.70–0.99)**
*p*-value		0.075	0.251		**0.017**	0.025		**0.047**	**0.044**
*p* for trend		0.308			0.085			0.068	
Loneliness									
Unadjusted model (PR; 95% CI)	Ref.	0.80 (0.59–1.09)	**0.64 (0.49–0.85)**	Ref.	**0.72 (0.56–0.93)**	**0.80 (0.66–0.98)**	Ref.	**0.75 (0.57–0.97)**	0.85 (0.66–1.08)
*p*-value		0.152	**0.003**		**0.012**	**0.030**		**0.033**	0.178
Adjusted model (PR; 95% CI)	Ref.	0.80 (0.61–1.06)	**0.61 (0.46–0.79)**	Ref.	**0.71 (0.56–0.89)**	**0.76 (0.64–0.91)**	Ref.	**0.72 (0.55–0.94)**	**0.77 (0.60–0.97)**
*p*-value		0.126	**0.001**		**0.005**	**0.004**		**0.017**	**0.034**
*p* for trend		**<0.001**			**0.005**			0.051	
Psychological stress									
Unadjusted model (PR; 95% CI)	Ref.	0.88 (0.69–1.12)	0.97 (0.81–1.16)	Ref.	**0.80 (0.67–0.96)**	1.00 (0.86–1.17)	Ref.	0.88 (0.75–1.02)	1.03 (0.85–1.25)
*p*-value		0.301	0.743		**0.017**	0.966		0.095	0.725
Adjusted model (PR; 95% CI)	Ref.	0.88 (0.72–1.09)	0.89 (0.76–1.03)	Ref.	**0.78 (0.65–0.93)**	0.91 (0.78–1.06)	Ref.	**0.83 (0.73–0.94)**	0.88 (0.74–1.05)
*p*-value		0.255	0.107		**0.007**	0.211		**0.005**	0.159
*p* for trend		0.119			0.258			0.226	
Suicidal ideation									
Unadjusted model (PR; 95% CI)	Ref.	1.01 (0.95–1.08)	1.01 (0.95–1.08)	Ref.	0.96 (0.91–1.02)	0.97 (0.91–1.03)	Ref.	0.98 (0.93–1.03)	1.02 (0.96–1.09)
*p*-value		0.741	0.674		0.236	0.335		0.390	0.497
Adjusted model (PR; 95% CI)	Ref.	0. 99 (0.94–1.06)	0.97 (0.91–1.04)	Ref.	0.95 (0.89–1.01)	**0.93 (0.87–0.99)**	Ref.	0.95 (0.90–1.00)	0.97 (0.90–1.04)
*p*-value		0.926	0.448		0.098	**0.046**		0.068	0.337
*p* for trend		0.441			0.047			0.391	

PR: prevalence ratio; CI: confidence interval. Poisson regression models adjusted for sociodemographic characteristics (age, sex, self-reported race/skin color, school shift, and family income), nutritional status, and lifestyle factors (physical activity, screen time). Values in bold indicate statistical significance (*p* < 0.05).

## Data Availability

The data that support the findings of this study are available from the author, GJSR, upon reasonable request.

## References

[1] Arias J.A., Williams C., Raghvani R., Aghajani M., Baez S., Belzung C., Booij L., Busatto G., Chiarella J., Fu C.H. (2020). The neuroscience of sadness: A multidisciplinary synthesis and collaborative review. Neurosci. Biobehav. Rev..

[2] Ma R., Mann F., Wang J., Lloyd-Evans B., Terhune J., Al-Shihabi A., Johnson S. (2019). The effectiveness of interventions for reducing subjective and objective social isolation among people with mental health problems: A systematic review. Soc. Psychiatry Psychiatr. Epidemiol..

[3] Epel E.S., Crosswell A.D., Mayer S.E., Prather A.A., Slavich G.M., Puterman E., Mendes W.B. (2018). More than a feeling: A unified view of stress measurement for population science. Front. Neuroendocrinol..

[4] De Leo D., Goodfellow B., Silverman M., Berman A., Mann J., Arensman E., Hawton K., Phillips M.R., Vijayakumar L., Andriessen K. (2021). International study of definitions of English-language terms for suicidal behaviours: A survey exploring preferred terminology. BMJ Open.

[5] McLaughlin K.A., Greif Green J., Gruber M.J., Sampson N.A., Zaslavsky A.M., Kessler R.C. (2012). Childhood adversities and first onset of psychiatric disorders in a national sample of US adolescents. Arch. Gen. Psychiatry.

[6] Vasan S., Lim M.H., Eikelis N., Lambert E. (2024). Investigating the relationship between early cardiovascular disease markers and loneliness in young adults. Sci. Rep..

[7] Pourriyahi H., Yazdanpanah N., Saghazadeh A., Rezaei N. (2021). Loneliness: An immunometabolic syndrome. Int. J. Environ. Res. Public Health.

[8] Wang J., Yang Y., Chen Y., Lin H., Wang T., Wang Z., Chen X., Fu C. (2024). Loneliness, internalizing and externalizing problems, and suicidal ideation among Chinese adolescents: A longitudinal mediation analysis. J. Adolesc. Health.

[9] Biswas T., Scott J.G., Munir K., Renzaho A.M., Rawal L.B., Baxter J., Mamun A.A. (2020). Global variation in the prevalence of suicidal ideation, anxiety and their correlates among adolescents: A population based study of 82 countries. eClinicalMedicine.

[10] Sauter S.R., Kim L.P., Jacobsen K.H. (2019). Loneliness and friendlessness among adolescents in 25 countries in Latin America and the Caribbean. Child Adolesc. Ment. Health.

[11] Instituto Brasileiro de Geografia e Estatística (IBGE) *Pesquisa Nacional de Saúde do Escolar: 2024*; IBGE: Rio de Janeiro, Brazil, 2026. https://biblioteca.ibge.gov.br/visualizacao/livros/liv102266.pdf.

[12] Silva F.M.d.A., Menezes A.S. (2018). Sedentary behavior, psychosocial stress indicators, and health-risk behaviors among adolescents in northeastern Brazil. J. Phys. Act. Health.

[13] Moya C.M., Soares F.C., Lima R.A., Júnior R.d.S.W., Aguilar J.A., Barros S.S.H., Küchler A.-M., de Barros M.V.G. (2026). Suicidal ideation and planning in adolescents: Temporal trends (2006–2022) and intersectional inequalities. Soc. Psychiatry Psychiatr. Epidemiol..

[14] de Oliveira M.F.B., de Castro I.R.R. (2022). Cooking autonomy: A multilevel conceptual model on healthy home cooking. Cad. Saude Publica.

[15] Dean M., O’KAne C., Issartel J., McCloat A., Mooney E., Gaul D., Wolfson J.A., Lavelle F. (2021). Guidelines for designing age-appropriate cooking interventions for children: The development of evidence-based cooking skill recommendations for children, using a multidisciplinary approach. Appetite.

[16] Selman S.B., Dilworth-Bart J.E. (2023). Routines and child development: A systematic review. J. Fam. Theory Rev..

[17] Farmer N., Touchton-Leonard K., Ross A. (2017). Psychosocial benefits of cooking interventions: A systematic review. Health Educ. Behav..

[18] Chen X., Li J., Zhang P., Hu H., Yan W., Peng K. (2024). Who experiences greater happiness when dining together? A study of the relationship between dining with family members and the mental health of Chinese students. Child Indic. Res..

[19] Elgar F.J., Craig W., Trites S.J. (2013). Family dinners, communication, and mental health in Canadian adolescents. J. Adolesc. Health.

[20] Berge J.M., MacLehose R.F., Larson N., Laska M., Neumark-Sztainer D. (2016). Family food preparation and its effects on adolescent dietary quality and eating patterns. J. Adolesc. Health.

[21] Dudley D.A., Cotton W.G., Peralta L.R. (2015). Teaching approaches and strategies that promote healthy eating in primary school children: A systematic review and meta-analysis. Int. J. Behav. Nutr. Phys. Act..

[22] Utter J., Denny S., Lucassen M., Dyson B. (2016). Adolescent cooking abilities and behaviors: Associations with nutrition and emotional well-being. J. Nutr. Educ. Behav..

[23] Castillo-Miñaca M.E., Mendoza-Gordillo M.J., Ruilova M., Yáñez-Sepúlveda R., Gutiérrez-Espinoza H., Olivares-Arancibia J., Andrade S., Ochoa-Avilés A., Tárraga-López P.J., López-Gil J.F. (2025). Is involvement in food tasks associated with psychosocial health in adolescents? The EHDLA study. Nutrients.

[24] Neves F.S., Martins C.A., Fontes V.S., Batista A.P., de Faria E.R., Netto M.P., Oliveira R.M.S., Cândido A.P.C. (2023). Involvement of Brazilian adolescents in home cooking: An exploratory analysis (EVA-JF Study). Nutrition.

[25] Lwanga S.K., Lemeshow S., Lwanga S.K., Lemeshow S., World Health Organization (1991). Sample Size Determination in Health Studies: A Practical Manual.

[26] Pinto A.d.A., Ribeiro G.J.S., Pelegrini A. (2025). Knowledge of physical activity guidelines and its association with meeting physical activity and sedentary behavior recommendations in adolescents. Children.

[27] Malta D.C., de Souza J.B., de Vasconcelos N.M., de Mello F.C.M., Buback J.B., Gomes C.S., Pereira C.A. (2024). Cyberbullying entre escolares brasileiros: Dados da Pesquisa Nacional de Saúde do Escolar, 2019. Cienc. Saude Coletiva.

[28] da Silva K.S., Lopes A.D.S., Hoefelmann L.P., Cabral L.G.d.A., De Bem M.F.L., de Barros M.V.G., Nahas M.V. (2013). Health risk behaviors project (COMPAC) in youth of the Santa Catarina State, Brazil: Ethics and methodological aspects. Rev. Bras. de Cineantropometria Desempenho Hum..

[29] Landis J.R., Koch G.G. (1977). The measurement of observer agreement for categorical data. Biometrics.

[30] World Health Organization (2026). BMI-for-Age (5–19 Years). https://www.who.int/tools/growth-reference-data-for-5to19-years/indicators/bmi-for-age.

[31] Bull F.C., Al-Ansari S.S., Biddle S., Borodulin K., Buman M.P., Cardon G., Carty C., Chaput J.-P., Chastin S., Chou R. (2020). World Health Organization 2020 guidelines on physical activity and sedentary behaviour. Br. J. Sports Med..

[32] Hirshkowitz M., Whiton K., Albert S.M., Alessi C., Bruni O., DonCarlos L., Hazen N., Herman J., Katz E.S., Kheirandish-Gozal L. (2015). National sleep foundation’s sleep time duration recommendations: Methodology and results summary. Sleep Health.

[33] Media C.O.C.A., Strasburger V.C., Hogan M.J., Mulligan D.A., Ameenuddin N., Christakis D.A., Cross C., Fagbuyi D.B., Hill D.L., Levine A.E. (2013). Children, Adolescents, and the Media. Pediatrics.

[34] Tepper D.L., Howell T.J., Bennett P.C. (2022). Executive functions and household chores: Does engagement in chores predict children’s cognition?. Aust. Occup. Ther. J..

[35] Li Y., Luo M., Wu X., Xiao Q., Luo J., Jia P. (2019). Grocery store access and childhood obesity: A systematic review and meta-analysis. Obes. Rev..

[36] Lantrip C., Isquith P.K., Koven N.S., Welsh K., Roth R.M. (2014). Executive function and emotion regulation strategy use in adolescents. Appl. Neuropsychol. Child.

[37] Hu W., Feng X., Luo S., Xu D., Xu Z., Chen W., Guo V.Y. (2025). Loneliness and self-reported executive functions in Chinese adolescents: The mediating role of subjective sleep quality. J. Affect. Disord..

[38] Wenzel S., Benkenstein M. (2018). Together always better? The impact of shopping companions and shopping motivation on adolescents’ shopping experience. J. Retail. Consum. Serv..

[39] Abeykoon A.H., Engler-Stringer R., Muhajarine N. (2017). Health-related outcomes of new grocery store interventions: A systematic review. Public Health Nutr..

[40] Sakız H., Mert A., Sarıçam H. (2020). Self-esteem and perceived social competence protect adolescent students against ostracism and loneliness. J. Psychol. Couns. Sch..

[41] Jonsson K.R., Bailey C.K., Corell M., Löfstedt P., Adjei N.K. (2024). Associations between dietary behaviours and the mental and physical well-being of Swedish adolescents. Child Adolesc. Psychiatry Ment. Health.

[42] Eisenberg M.E., Olson R.E., Neumark-Sztainer D., Story M., Bearinger L.H. (2004). Correlations between family meals and psychosocial well-being among adolescents. Arch. Pediatr. Adolesc. Med..

[43] LeBlanc J., Ward S., LeBlanc C.P. (2022). The association between adolescents’ food literacy, vegetable and fruit consumption, and other eating behaviors. Health Educ. Behav..

[44] Vaitkeviciute R., E Ball L., Harris N. (2014). The relationship between food literacy and dietary intake in adolescents: A systematic review. Public Health Nutr..

[45] Leal F.M.d.R., de Oliveira B.M.P.M., Pereira S.S.R. (2011). Relationship between cooking habits and skills and Mediterranean diet in a sample of Portuguese adolescents. Perspect. Public Health.

[46] Fismen A.-S., Aarø L.E., Thorsteinsson E., Ojala K., Samdal O., Helleve A., Eriksson C. (2024). Associations between eating habits and mental health among adolescents in five nordic countries: A cross-sectional survey. BMC Public Health.

[47] Wong P.T., Barnes K., Kirkegaard A., Lepre B., Bartrim K., Wright O., Crowley J., Sullivan V., Ball L. (2025). The Impact of Culinary Education on Adolescents’ Psychosocial Wellbeing: A Systematic Review. J. Community Appl. Soc. Psychol..

